# TSLP pretreatment inhibits M1 macrophage polarization and attenuates LPS-induced iNKT cell-dependent acute lung injury

**DOI:** 10.3389/fimmu.2025.1583235

**Published:** 2025-05-23

**Authors:** Ting Zhou, Ziyao Zhang, Yawen Zhan, Meiying Wang, Mi Wu, Xiufang Weng, Younian Xu

**Affiliations:** ^1^ Department of Immunology, School of Basic Medicine, Tongji Medical College, Huazhong University of Science and Technology, Wuhan, China; ^2^ Department of Anesthesiology, Union Hospital, Tongji Medical College, Huazhong University of Science and Technology, Wuhan, China; ^3^ Institute of Anesthesia and Critical Care Medicine, Union Hospital, Tongji Medical College, Huazhong University of Science and Technology, Wuhan, China; ^4^ Key Laboratory of Anesthesiology and Resuscitation (Huazhong University of Science and Technology), Ministry of Education, Wuhan, China; ^5^ Key Laboratory of Organ Transplantation, Ministry of Education, Wuhan, China; ^6^ National Health Commission (NHC), Key Laboratory of Organ Transplantation, Wuhan, China; ^7^ Key Laboratory of Organ Transplantation, Chinese Academy of Medical Sciences, Wuhan, China

**Keywords:** ARDS, acute lung injury, TSLP, macrophage, iNKT cells

## Abstract

**Introduction:**

Sepsis associated acute respiratory distress syndrome (ARDS), is a life-threatening condition characterized by severe pulmonary inflammation. Previous research has suggested that allergic immune diseases are associated with a lower risk of sepsis. Therefore, we hypothesized that certain molecules involved in type 2 inflammation are beneficial for the outcome of sepsis associated ARDS. Thymic stromal lymphopoietin (TSLP) is known to promote Th2 responses in allergic disease, however, its role in sepsis associated ARDS remains limited.

**Methods:**

To investigate the role of TSLP in sepsis associated lung injury, we administered exogenous recombinant TSLP to wild-type mice, followed by lipopolysaccharide (LPS) challenge. At 24 hours post-treatment, bronchoalveolar lavage fluid (BALF) and lung tissues were collected for analysis. The ratio, number, phenotype, and function of immune cells and cytokine levels were measured. Additionally, murine bone marrow-derived macrophages (BMDMs) were prepared and stimulated with LPS and TSLP to further verify our findings experimentally. To explore the molecular mechanisms of TSLP’s effect, analysis of transcriptome sequencing and single-cell transcriptome sequencing and subsequent experiments were performed.

**Results:**

In LPS-induced acute lung injury models, pretreatment with TSLP significantly alleviated lung injury, suppressed inflammatory cytokines secretion, and reduced macrophages and neutrophils infiltration. In addition, TSLP treatment significantly inhibited M1 macrophage polarization and promoted M2 macrophage differentiation. Transcriptome sequencing suggested IFN-γ as a potential target of TSLP, and single-cell transcriptome sequencing showed that innate like T cells are important source of IFN-γ. Consistently, flow cytometry showed that proportion of IFN-γ-producing iNKT cells was decreased by TSLP administration in the acute lung injury model. Intriguingly, Jα18^−/−^ mice, which are completely deficient in invariant natural killer T (iNKT) cells, exhibited not only significantly less severe lung inflammation but also a notably higher degree of anti-inflammatory Arg1^+^ M2 macrophages infiltration when compared with their LPS-sensitized wild-type counterparts.

**Conclusions:**

These findings not only underscore the crucial role of TSLP in the regulation of sepsis-associated ARDS but also demonstrate its potential clinical value as both a predictive biomarker for early detection and a molecular target for therapeutic intervention.

## Introduction

Acute respiratory distress syndrome (ARDS), characterized by hypoxemia, pulmonary edema, and profound interconnected inflammatory cascades, is accompanied by high mortality rates and unfavorable outcomes (1). The onset of ARDS typically involves a normal immune response to infection or injury, followed by Th1-cytokines release and excessive activation of inflammatory cells (2), especially proinflammatory macrophage activation (3). As one of the most common etiologies of ARDS, sepsis is closely associated with the development of ARDS and can cause multi-organ impairment. The lung tissues are the most susceptible to the attack of sepsis, thus, patients with sepsis are more likely to develop into acute lung injury or ARDS (4). Currently, anti-inflammatory pharmacologic interventions have not demonstrated significant curative effects and lung-protective mechanical ventilation remains the primary treatment (5).

Recent research has suggested that asthma and other allergic immune diseases are associated with a lower risk of sepsis or pneumonia (6–8). The mechanisms underlying the phenomenon remains unclear, and several factors may act in concert to the outcomes, such as age of allergy onset and a protective shielding to outer environmental exposure (7).

Thymic stromal lymphopoietin (TSLP) has garnered significant attention from researchers due to its role in promoting Th2 responses in allergic disease. It has been approved as a therapeutic target for asthma (9). Structurally similar to IL-7, TSLP shares the receptor constituent part IL-7Rα with IL-7 (10). TSLP not only induces proallergic CD4 T cell responses via dendritic cells but also promotes the polarization of M2 macrophage and the secretion of IL-4 and IL-13 from iNKT cells, thereby amplifying allergic inflammation (11, 12). Given these functions, we hypothesized that pre-existing type 2 immune response cytokines, such as TSLP, may confer benefits in the context of sepsis-associated ARDS.

In the present study, we elucidated that pretreatment with TSLP alleviates lung inflammation and diminishes the infiltration of pro-inflammatory neutrophils and M1 macrophages in an LPS- induced acute lung injury model. We identified IFN-γ as a potential target of TSLP, and administration of TSLP led to a reduction in IFN-γ-producing iNKT cells in this model. Collectively, our findings highlight the crucial role of TSLP in immune regulation of sepsis-associated ARDS and demonstrate its clinical potential clinical value as both a predictive biomarker and therapeutic target.

## Methods

### Animal studies and ethics statement

C57BL/6J wild-type male mice were purchased from SJA Laboratories (China), and Jα18^-/-^ male mice with a B6 background were kindly provided by Prof. Li Bai. All the mice were housed under specific pathogen-free conditions (12/12 h light/dark cycle, 55% ± 5% humidity, 24°C). The acute lung injury model was induced by intranasal instillation of LPS (from *E. coli* O111: B4, LPS25, Sigma, USA) at a dose of 5 mg/kg after 6 h of pretreatment with recombinant TSLP (TSLP) (HY-P70626, MCE, China) or an isopycnic vehicle intranasally. Eight-week-old wild-type mice were randomly assigned to one of the following four groups: vehicle control, LPS alone, TSLP 2.5 μg/kg+ LPS, and TSLP 10 μg/kg + LPS. Mice were euthanized 24 hours after LPS inhalation, and lung tissues and bronchoalveolar lavage fluid (BALF) were harvested. BALF was obtained by gently flushing the bronchoalveolar cavity with 1 ml of precooled PBS, repeated three times.

All animal experiments were approved by the Experimental Animal Ethics Committee of Huazhong University of Science and Technology (IACUC: S4517), and animal care was conducted in accordance with the institutional guidelines.

### H&E staining

The lung tissues were fixed in 4% paraformaldehyde (BL539A, Biosharp, China), embedded in paraffin, and sliced into 5-μm-thick sections. After deparaffinization, the sections were stained with hematoxylin–eosin and scanned via Pannoramic MIDI (3D HISTECH). The level of lung injury was assessed by adhering to blinding requirements according to the Smith score as previously described (13).

### Single-cell suspension preparation

Single-cell suspensions of mouse lung tissue were prepared as previously described (14). Briefly, lung tissues were perfused with PBS via the right ventricle, minced into pieces, and then digested in a solution containing Liberase TL (05401020001, Sigma) and DNase I (10104159001, Sigma) at 37 °C for 30 min. The cell suspensions were then passed through a 70 μm cell strainer, washed, and resuspended in precooled PBS supplemented with 1% FBS (A5256701, Gibco, USA). Lymphocytes were isolated by density gradient centrifugation using Percoll (17089101, Cytiva). Meanwhile, BALF was centrifuged to separate the cells from supernatants.

### Flow cytometry

Prior to staining with antibodies, cells were blocked with an anti-CD16/32 antibody (clone 93; BioLegend, USA). Fluorescence-conjugated antibodies against the following markers were obtained from BD Biosciences (USA), eBioscience (USA), and BioLegend: anti-CD45 (30-F11), anti-TCR-β (H57-597), anti-CD4 (RM4-5), anti-CD8 (53-6.7), anti-CD11b (M1/70), anti-Ly6G (1A8), anti-F4/80 (BM8), anti-iNOS (CXNFT), anti-ARG1 (A1exF5), anti-IL-4 (11B11) and anti-IFN-γ (XMG1.2). Viability was assessed using the Zombie Fixable Viability Kit (BioLegend, USA). Fluorescence -conjugated mCD1d/PBS-57 tetramers were acquired from the National Institutes of Health (NIH) tetramer facility for iNKT staining. For intracellular IFN-γ staining, isolated cells were stimulated with phorbol myristate acetate (PMA) (25 ng/ml, P8139, Sigma) and ionomycin (Ion) (500 ng/ml, 407953, Sigma) for 0.5 h, followed by incubation with brefeldin A (00-4506-51, Invitrogen, USA) for an additional 3.5 h. Multiple cytokine levels in the BALF or culture supernatant of bone marrow-derived macrophages (BMDMs) were measured using the LEGENDplex Multi-Analyte Flow Assay Kit (740446, BioLegend, USA)). Data were collected on a FACSVerse cytometer (BD Biosciences) and analyzed using FlowJo software (Tree Star).

### Isolation and culture of BMDMs

Murine bone marrow cells were obtained from the femur and tibia of C57BL/6J male mice according to the protocol described by Toda et al. (15). The mice were first euthanized, and their lower limbs were carefully exposed to facilitate the collection the femurs and tibias. The bone marrow was then flushed from these bones. This suspension was subsequently centrifuged at 200 × g for 5 min at 4°C. The resulting cell pellet was resuspended in a lysis buffer to selectively remove erythrocytes. Following erythrocyte lysis, the cells were washed with PBS and cultured in RMPI medium supplemented with 10% FBS (A5256701, Gibco, USA), 1% penicillin–streptomycin (PB180120, Procell, China), and 50 ng/ml recombinant macrophage colony-stimulating factor (M-CSF, 315-02, Peprotech, USA) at 37°C with 5% CO2 for 7 days. Then, the BMDMs were harvested and stimulated with TSLP (5 ng/ml) and LPS (100 ng/ml).

### Quantitative real-time polymerase chain reaction

Total cellular RNA was extracted using TRIzol Reagent (21101, Agbio, China) and reverse-transcribed into cDNA using a first-strand cDNA synthesis kit (Vazyme, China) according to the manufacturer’s instructions. Quantitative real-time PCR was then performed using TB Green Premix Ex Taq (RR420A, Takara, Japan) with a CFX96 Real-Time PCR Detection System (Bio-Rad, USA). The relative expression levels of the target genes were normalized to the expression level of the housekeeping gene *Actb*. The sequences of the qPCR primers used were as follows: *Actb* forward, 5-GGCTGTATTCCCCTCCATCG-3; *Actb* reverse, 5-CCAGTTGGTAACAATGCCATGT-3; *Nos2* forward, 5-GCTGCCAGGGTCACAACTT-3; *Nos2* reverse, 5-AACAGCTCAGTCCCTTCACC-3; *Chil3* forward, 5-TACTATGAGGCTCAGTGGC-3; and *Chil3* reverse, 5-ACAGAAAGAACCACTGAAGTC-3; *Arg1* forward, 5-GAACTGAAAGGAAAGTTCCCA-3; and *Arg1* reverse, 5-AATGTACACGATGTCTTTGGC-3.

### RNA- sequencing and single-cell RNA sequencing

In brief, the total RNA was extracted from the lung tissues of mice treated with vehicle alone (Veh), LPS alone (LPS group), or TSLP (10 μg/kg) +LPS (LPS+TSLP). The quantity and quality of the extracted RNA were assayed using an Agilent 2100 bioanalyzer (Agilent Technologies, USA). Subsequently, poly(A)+ mRNA was isolated using Oligo(dT) magnetic beads. First-strand cDNA synthesis was then performed followed by purification. For library construction targeting the 3’-end of polyadenylated transcripts, the NEBNext Ultra RNA Library Prep Kit for Illumina (E7530, New England Biolabs, USA) was employed according to the manufacturer’s protocol. Quality assessment of final libraries was conducted using two complementary approaches: DNA concentration quantification via Qubit 2.0 Fluorometer (Thermo Fisher Scientific,USA) and size distribution analysis with Agilent 2100 Bioanalyzer (Agilent Technologies, USA). The libraries were sequenced using a NovaSeq 6000 (Illumina, USA). Raw sequencing data were filtered to remove low-quality reads prior to sequence alignment and gene expression quantification. Principal component analysis was performed using the “prcomp” function from the “ggplot2” (3.4.3) package. Differential gene expression analysis was conducted using the “Deseq2” (1.40.2) package, with genes exhibiting a Log2FoldChange>1 or <-1 and a P value<0.05 considered differentially expressed. The results were visualized via volcano plots generated using the “ggpubr” (0.6.0) package. Gene set enrichment analysis (GSEA) and GSEA based on gene ontology (GO) database (GSEA-GO) analysis were performed using the “clusterProfiler” (4.8.2) package. Enrichment score (NES) with a normalized enrichment score (NES) greater than 1 or less than -1 and a P value lower than 0.05 were considered significant. The Bulk RNA-seq dataset GSE262107 is available in the NCBI Gene Expression Omnibus (GEO) database.

To analyze the expression of *Ifng* in immune cell populations, the scRNA-seq dataset GSE167118, containing three patients with bacterial pneumonia-induced ARDS, was obtained from GEO datasets (https://www.ncbi.nlm.nih.gov/). Computational analysis was performed using the “Seurat” (4.3.0.1) package. Gene expression patterns were visualized via the R package “pheatmap” (1.0.12) and the Seurat “DotPlot” function.

### Statistical analysis

Statistical analyses were conducted using GraphPad Prism version 8.0. Comparisons between two groups were analyzed using either unpaired Student’s *t* test (for data with normal distribution and equal variance) or the Mann–Whitney test (for non-normally distributed variables). For comparisons involving multiple groups, one-way ANOVA was employed for data with normal distribution and equal variance and Kruskal-Wallis test was employed for non-normally distributed variables. Data are presented as means ± SEMs, with each experiment performed at least in triplicate. A P value of less than 0.05 was considered statistically significant.

## Results

### TSLP alleviates lung injury and reduces inflammatory cytokine secretion in LPS-induced acute lung injury

**Figure 1:**
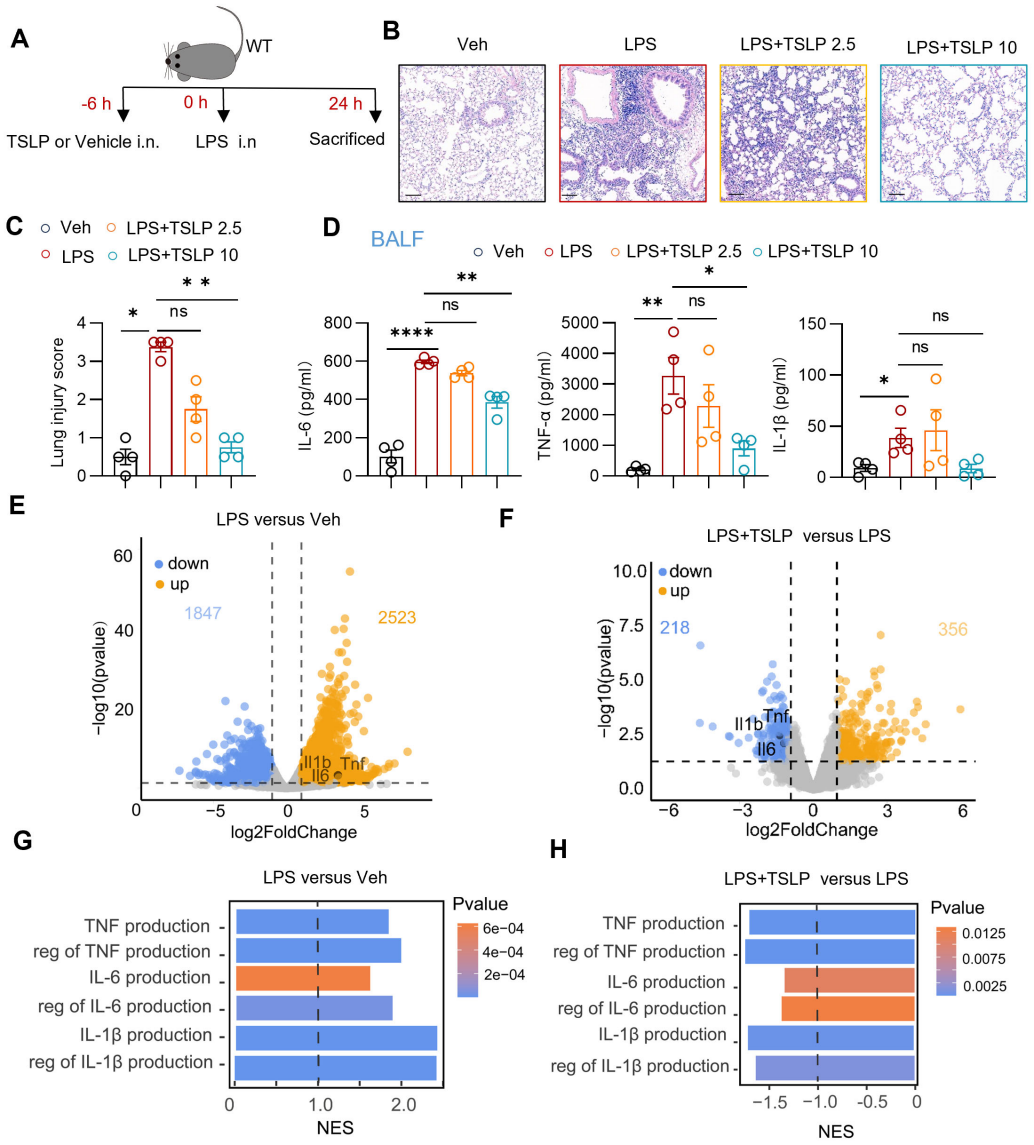
TSLP alleviated lung inflammation in LPS-induced acute lung injury. **(A)** The flow chart showing the process of the experimental animal studies. **(B, C)** Representative sections and lung injury score evaluation of H&E staining. Scale bar = 100 μm. n = 4 per group, Mann Whitney test for statistics analysis between Veh group and LPS group, Kruskal-Wallis test for statistics analysis between LPS group, LPS+TSLP 2.5 group, LPS+TSLP 10 group. **(D)** Summary bar graphs with scatter plots showing the concentration of IL-6, TNF-α, and IL-1β in the BALF of groups treated with Vehicle, LPS, LPS+ 2.5 μg/kg TSLP (LPS+TSLP 2.5), and LPS+ 10 μg/kg TSLP (LPS+TSLP 10). n = 4 per group, for IL-6, unpaired Student’s t test for statistics analysis between Veh group and LPS group, Kruskal-Wallis test for statistics analysis between LPS group, LPS+TSLP 2.5 group, LPS+TSLP 10 group. For TNF-α and IL-1β, unpaired Student’s t test for statistics analysis between Veh group and LPS group, one-way ANOVA test for statistics analysis between LPS group, LPS+TSLP 2.5 group, LPS+TSLP 10 group. **(E)** The volcano plot showing differentially expressed genes of the lung tissues of LPS group versus Veh group. **(F)** The volcano plot showing differentially expressed genes of the lung tissues of LPS+TSLP group versus LPS group. **(G)** The bar chart of indicated enriched GO pathways in the lung tissues of LPS group versus Veh group **(H)** The bar chart of indicated enriched GO pathways in the lung tissues of LPS+TSLP group versus LPS group. *P < 0.05, **P < 0.01, ****P < 0.0001, ns, no significance. TSLP, Thymic Stromal Lymphopoietin, LPS, Lipopolysaccharide, Veh, Vehicle, BALF, Broncho-alveolar lavage fluid, WT, wild type, NES, normalized enrichment score. Data are represented as mean ± SEM.

LPS was administered nasally to C57BL/6J mice to simulate sepsis-associated acute lung injury as previously described (16). To explore the effect of TSLP in this acute lung injury model, wild-type mice were pretreated with TSLP via intranasal administration (**Figure 1A**). Histological examination by H&E staining revealed significant alveolar septal thickening and infiltration of inflammatory cells in the lung tissues of LPS-treated mice. These pathological changes were markedly attenuated by pretreatment with either 2.5 μg/kg or 10 μg/kg TSLP (**Figure 1B**). The lung injury score, which reflects the degree of inflammation-mediated injury, was decreased in the TSLP-treated groups compared to the LPS alone group (**Figure 1C**). Notably, pre-treatment with 10 μg/kg TSLP alone did not induce any significant histological alterations compared to vehicle administration (**Supplementary Figure 1A**). Furthermore, the concentrations of proinflammatory cytokines, including IL-6, TNF-α and IL-1β, were significantly elevated in the BALF of LPS-treated mice, but IL-6 and TNF-α were substantially reduced upon pretreatment with10 μg/kg TSLP (**Figure 1D**).

Principal component analysis of the transcriptomic data revealed distinct transcriptional signatures in lung tissue between the vehicle-treated (Veh) group (n=3) and LPS-treated (LPS) group (n=3), as well as between the 10 μg/kg TSLP-pretreated (LPS+TSLP) group (n=4) and the LPS group (**Supplementary Figure 1B**). In line with the protein expression changes, transcripts of *Il6*, *Tnf*, and *Il1b* were significantly upregulated in the lung tissue of the LPS group compared with the Veh group (**Figure 1E**), and were downregulated in the LPS+TSLP group compared to the LPS group (**Figure 1F**). Correspondingly, GSEA-GO analysis revealed that pathways related to the productions of cytokines (IL-6, TNF-α and IL-1β) in the lung tissue of LPS group were upregulated compared to the Veh group (**Figure 1G**). In contrast, these pathways were downregulated in the LPS + TSLP group compared to the LPS group (**Figure 1H**). Collectively, our findings indicate that TSLP exerts a protective effect against sepsis-associated acute lung injury by reducing lung tissue damage and suppressing the secretion of inflammatory cytokines.

### Overview of immune cell infiltration during LPS exposure with or without TSLP pretreatment

**Figure 2:**
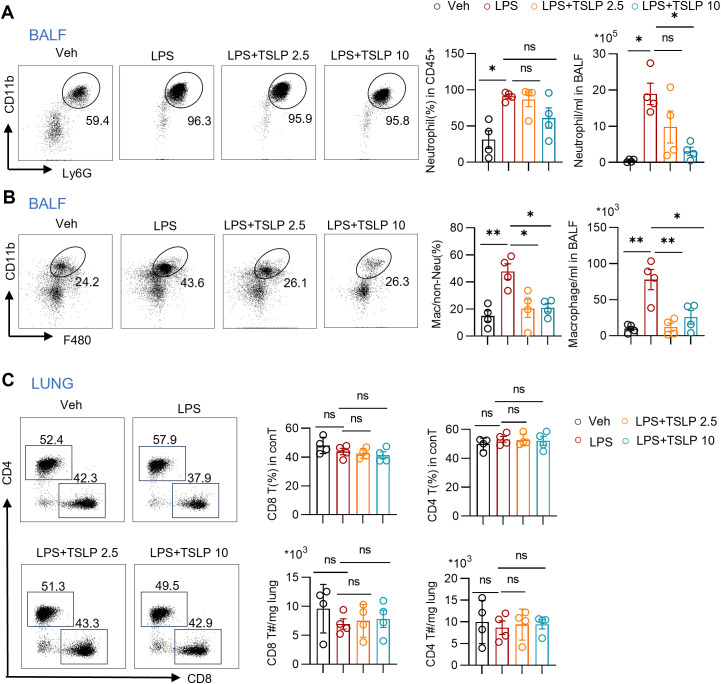
Immune cell overview during LPS exposure with or without TSLP pretreatment. **(A)** Representative histogram and summary bar graphs with scatter plots showing neutrophil ratio and count in BALF of indicated groups. n = 4 per group, for neutrophil ratio, unpaired Student’s t test for statistics analysis between Veh group and LPS group, Kruskal-Wallis test for statistics analysis between LPS group, LPS+TSLP 2.5 group, LPS+TSLP 10 group. For neutrophil count, unpaired Student’s t test for statistics analysis between Veh group and LPS group, one-way ANOVA test for statistics analysis between LPS group, LPS+TSLP 2.5 group, LPS+TSLP 10 group. **(B)** Representative histogram and summary bar graphs with scatter plots showing ratio and count of macrophages in BALF of indicated groups. n = 4 per group, unpaired Student’s t test for statistics analysis between Veh group and LPS group, one-way ANOVA test for statistics analysis between LPS group, LPS+TSLP 2.5 group, LPS+TSLP 10 group. **(C)** Representative histogram and summary bar graphs with scatter plots showing ratio and counts of CD4 T cells and CD8 T cells in lung tissues of indicated groups. n = 4 per group, for ratio and counts of CD8 T cells and ratio of CD4 T cells, unpaired Student’s t test for statistics analysis between Veh group and LPS group, one-way ANOVA test for statistics analysis between LPS group, LPS+TSLP 2.5 group, LPS+TSLP 10 group. For counts of CD4 T cells, unpaired Student’s t test for statistics analysis between Veh group and LPS group, Kruskal-Wallis test for statistics analysis between LPS group, LPS+TSLP 2.5 group, LPS+TSLP 10 group. *P < 0.05, **P < 0.01, ns, no significance. TSLP, Thymic Stromal Lymphopoietin, LPS, Lipopolysaccharide, Veh, Vehicle, BALF, Broncho-alveolar lavage fluid. conT, conventional T cells, T#, T cell number. Data are represented as mean ± SEM.

We subsequently investigated the infiltration of neutrophils, macrophages, and T cells in the BALF and lung tissue of acute lung injury model, and assessed the impacts of TSLP. The gating strategies for CD11b^+^Ly6G^+^ neutrophils, CD11b^+^F480^+^ macrophages, CD8 T cells and CD4 T cells are presented in the **Supplementary Figures 2A**, **B**. Compared with those in the vehicle-treated group, the numbers and ratios of both neutrophils and macrophages, key players in the inflammatory cascade, in the BALF of mice following LPS treatment were significantly increased (**Figures 2A, B**). Pretreatment with TSLP, especially at a dose of 10 μg/kg, attenuated the increase in neutrophil and macrophage infiltration (**Figures 2A, B**). In contrast, no significant differences in the populations of CD8 T or CD4 T cells were observed in the lung tissue from LPS group compared with the Veh group, and TSLP treatment did not affect the ratio or number of these T cell subsets (**Figure 2C**). Collectively, these findings indicate that TSLP pretreatment significantly alleviates macrophage and neutrophil infiltration, but has no impact on conventional T cells.

### TSLP suppresses M1 macrophage polarization

**Figure 3:**
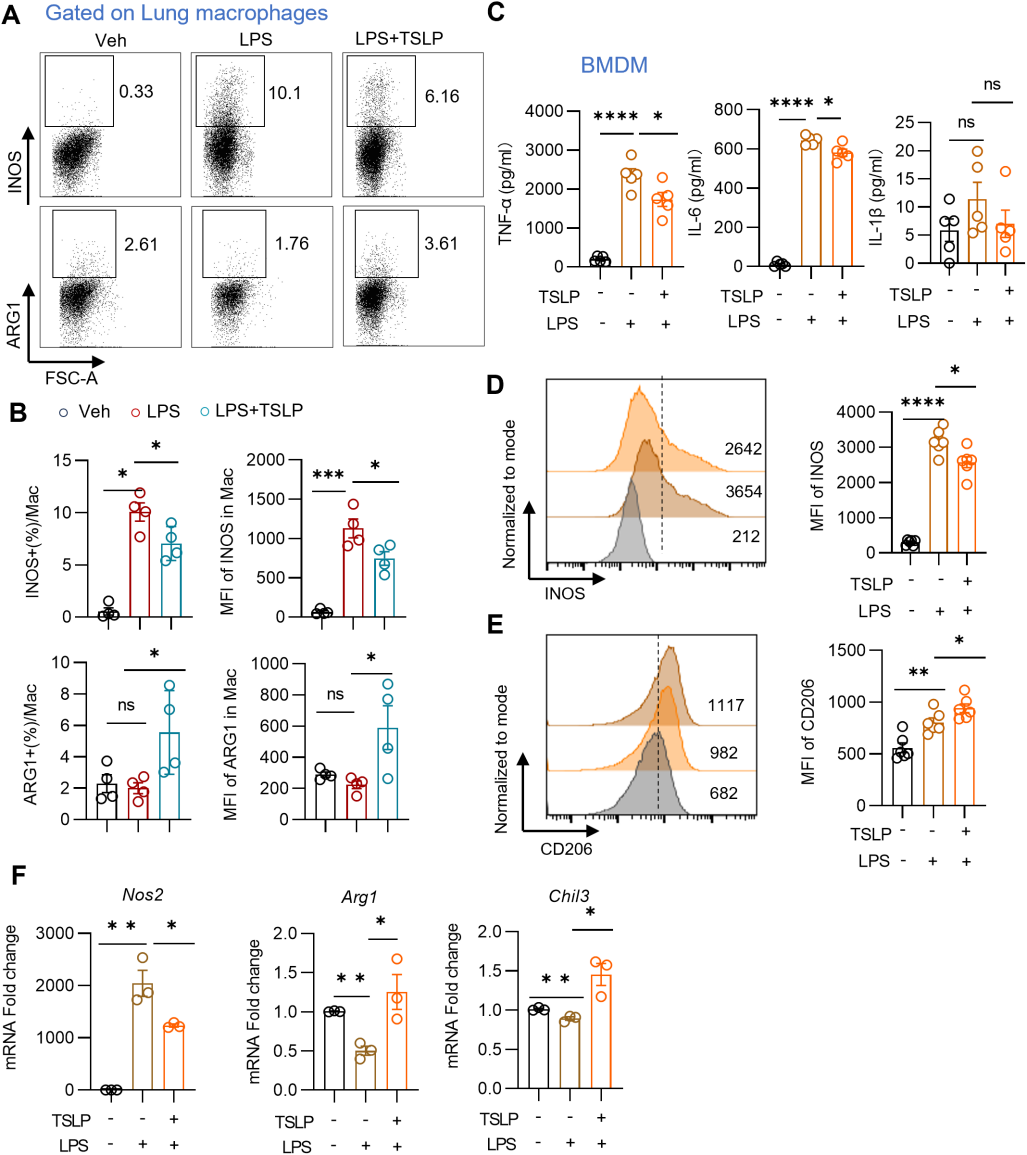
TSLP reduces M1 macrophages polarization *in vivo* and *in vitro*. **(A, B)** Representative histogram and summary bar graph with scatter plots showing INOS^+^ or ARG1^+^ pulmonary macrophages in indicated groups. n = 4 per group, for ratio of INOS, Mann Whitney test for statistics analysis between Veh group and LPS group, unpaired Student’s t test for statistics analysis between LPS group and LPS+TSLP group. For ratio of ARG1, MFI of INOS and ARG1, unpaired Student’s t test for statistics analysis between Veh group and LPS group or LPS group and LPS+TSLP group. **(C)** Summary bar graphs with scatter plots showing the concentration of TNF-α, IL-6, and IL-1β in the supernatant of BMDM in indicated groups. n = 5 per group, unpaired Student’s t test for statistics analysis between Veh group and LPS group or LPS group and LPS+TSLP group. **(D, E)** Representative histogram and summary bar graph with scatter plots showing INOS and CD206 expression in BMDM in indicated groups. n = 6 per group, unpaired Student’s t test for statistics analysis between Veh group and LPS group or LPS group and LPS+TSLP group. **(F)** Summary bar graph with scatter plots shows *Nos2*, *Arg1* and *Chil3* mRNA foldchange in BMDM in indicated groups. n = 3 per group, unpaired Student’s t test for statistics analysis between Veh group and LPS group or LPS group and LPS+TSLP group. *P < 0.05, **P < 0.01, ***P < 0.001, ****P < 0.0001, ns, no significance, TSLP, Thymic Stromal Lymphopoietin, LPS, Lipopolysaccharide, Veh, Vehicle, BMDM, bone marrow-derived macrophages, mac, macrophages. Data are represented as mean ± SEM.

Proinflammatory M1 macrophage have been identified as critical contributors to neutrophil migration in ARDS (17). In order to investigate the effects of TSLP on macrophage polarization in ARDS, we examined the ratios of proinflammatory M1 and anti-inflammatory M2 subpopulations among macrophages in the context of LPS treatment, both *in vivo* and *in vitro*. Following TSLP pretreatment, pulmonary macrophages (the gating strategy shown in **Supplementary Figure 2A**) exhibited reduced expression of INOS, a canonical marker of the proinflammatory M1 phenotype, and increased expression of ARG1, a marker of the M2 phenotype. This was evidenced by the analysis of the ratio and mean fluorescence intensity (MFI) (**Figures 3A, B**). These results suggest that TSLP promotes M2 polarization in acute lung injury model. To further verify the impact of TSLP on macrophage polarization, we exposed BMDMs from wild-type B6 mice to LPS, with or without 6-hour TSLP pretreatment. After 24 hours of incubation with LPS, cytokines in the culture supernatant were detected via flow cytometry. TSLP treatment significantly inhibited the secretion of IL-6 and TNF-α in response to LPS stimulation, although no significant trend was observed in IL-1β expression levels (**Figure 3C**). Additionally, TSLP exposure reduced the protein expression of INOS and increased the levels of CD206 in LPS-treated BMDMs (**Figures 3D, E**). Consistently, the increased mRNA expression of *Nos2* and the reduced mRNA expression of *Arg1* and *Chil3*-another marker of M2 macrophages- induced by LPS were reversed by TSLP pretreatment (**Figure 3F**). In summary, our findings indicate that TSLP suppresses M1 macrophage polarization both *in vivo* and *in vitro*, which may explain the attenuation of pulmonary inflammation by TSLP.

### TSLP downregulates IFN-γ related pro-inflammatory responses and suppresses IFN-γ-producing iNKT cells

**Figure 4:**
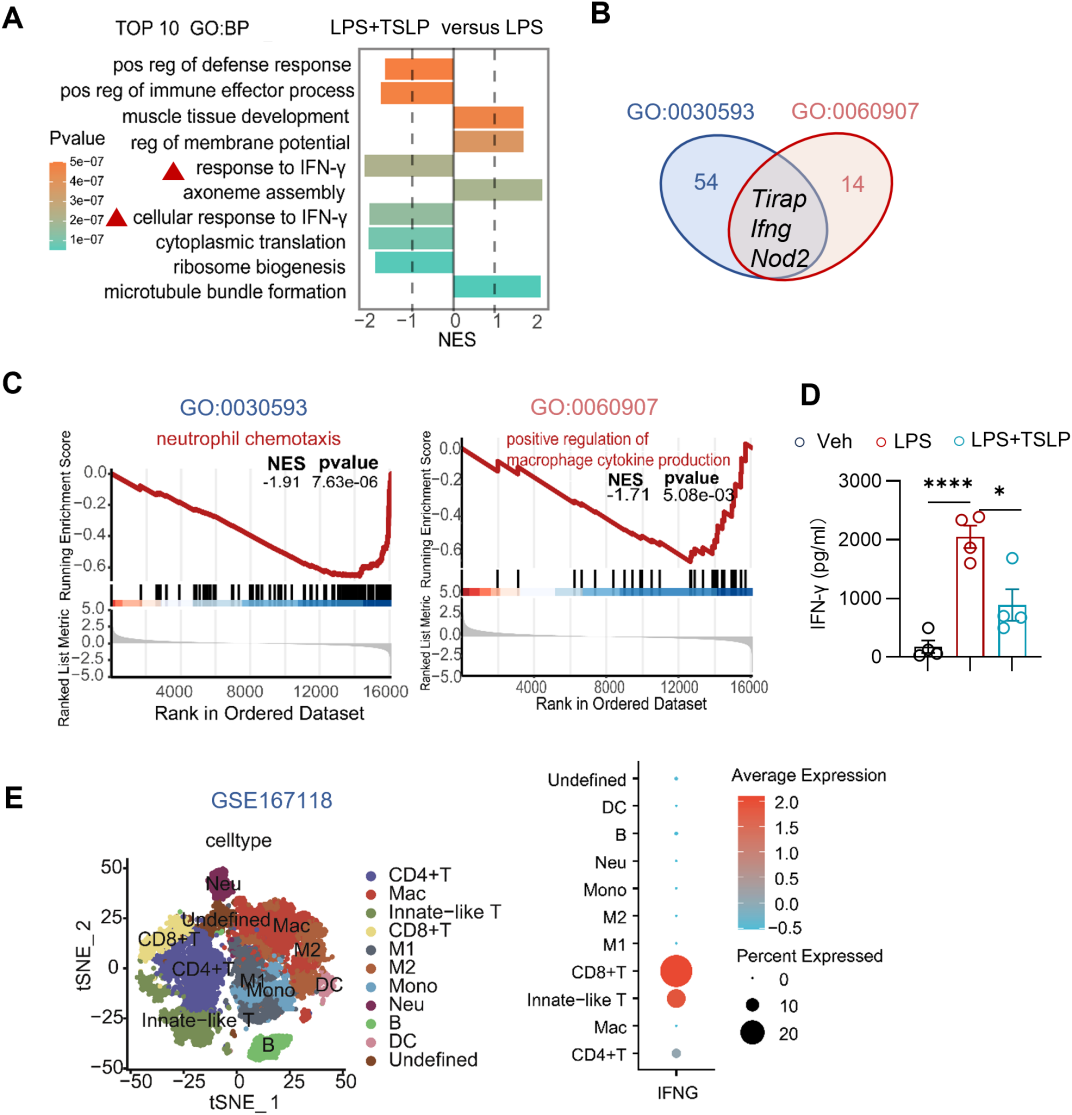
Exogenous TSLP notably downregulates IFN-γ-related pro-inflammatory responses in LPS-induced acute lung injury. **(A)** The plot showing NES values of the top 10 significantly altered biological processes (NES>1 or <-1, P value<0.05) from GSEA-GO analysis ranged by P value and the colors representing P value. **(B)** Venn diagram shows core enrichment genes of both neutrophil chemotaxis pathway (GO:0030593) and positive regulation of macrophage cytokines production (GO: 0060907). **(C)** GSEA plots for neutrophil chemotaxis pathway (GO:0030593) and positive regulation of macrophage cytokines production (GO: 0060907) biological process in LPS +TSLP group versus LPS group. **(D)** Summary bar graph with scatter plots depicting BALF IFN-γ concentration from indicated groups. n = 4 per group, unpaired Student’s t test for statistics analysis between Veh group and LPS group or LPS group and LPS+TSLP group. **(E)** tSNE plot of scRNAseq dataset (GSE167118) showing main clusters and Dotplot showing *IFNG* gene expression in immune cells. *P < 0.05, ****P < 0.0001. TSLP, Thymic Stromal Lymphopoietin, LPS, Lipopolysaccharide, Veh, Vehicle, GO, Gene ontology, NES, normalized enrichment score, BP, biological process, mac, macrophages, Neu, neutrophil, Mono, monocyte. Data are represented as mean ± SEM.

Further analysis revealed that pathways associated with the response to IFN-γ and cellular responses to IFN-γ ranked among the top 10 significantly altered biological processes in the lung tissue of the LPS+TSLP group compared to the LPS group. Notably, a negative NES lower than -1 suggested downregulation of IFN-γ-related responses in the TSLP group (**Figure 4A**). Moreover, the gene *Ifng* was found to be enriched in both the neutrophil chemotaxis pathway (GO: 0030593) and the positive regulation of macrophage cytokine production pathway (GO: 0060907) (**Figure 4B**), both of which were downregulated in the lung tissue of the LPS+TSLP group (n=4) compared to the LPS group (n=3) (**Figure 4C**). These results suggest that TSLP pretreatment inhibits IFN-γ-related responses crucial for neutrophil infiltration and macrophage-mediated inflammation. Additionally, TSLP consistently mitigated LPS-induced IFN-γ secretion in the BALF (**Figure 4D**). To further elucidate the cellular sources of *Ifng*, we performed a secondary analysis of a published single-cell RNA sequencing datasets from patients with bacterial pneumonia-induced ARDS (GSE167118) (18). Macrophage subsets, various T cell subpopulations, and neutrophils identified by marker genes were enriched in the BALF cells of these patients (**Figure 4E** and **Supplementary Figure 3A**). Notably, the *IFNG* transcript was predominantly distributed in CD8 T cells, innate-like T cells, and CD4 T cells (**Figure 4E**). These findings suggest that the protective effect of TSLP in inhibiting neutrophil chemotaxis and macrophage-associated proinflammatory cytokine production is probably associated with IFN-γ in acute lung injury.

**Figure 5:**
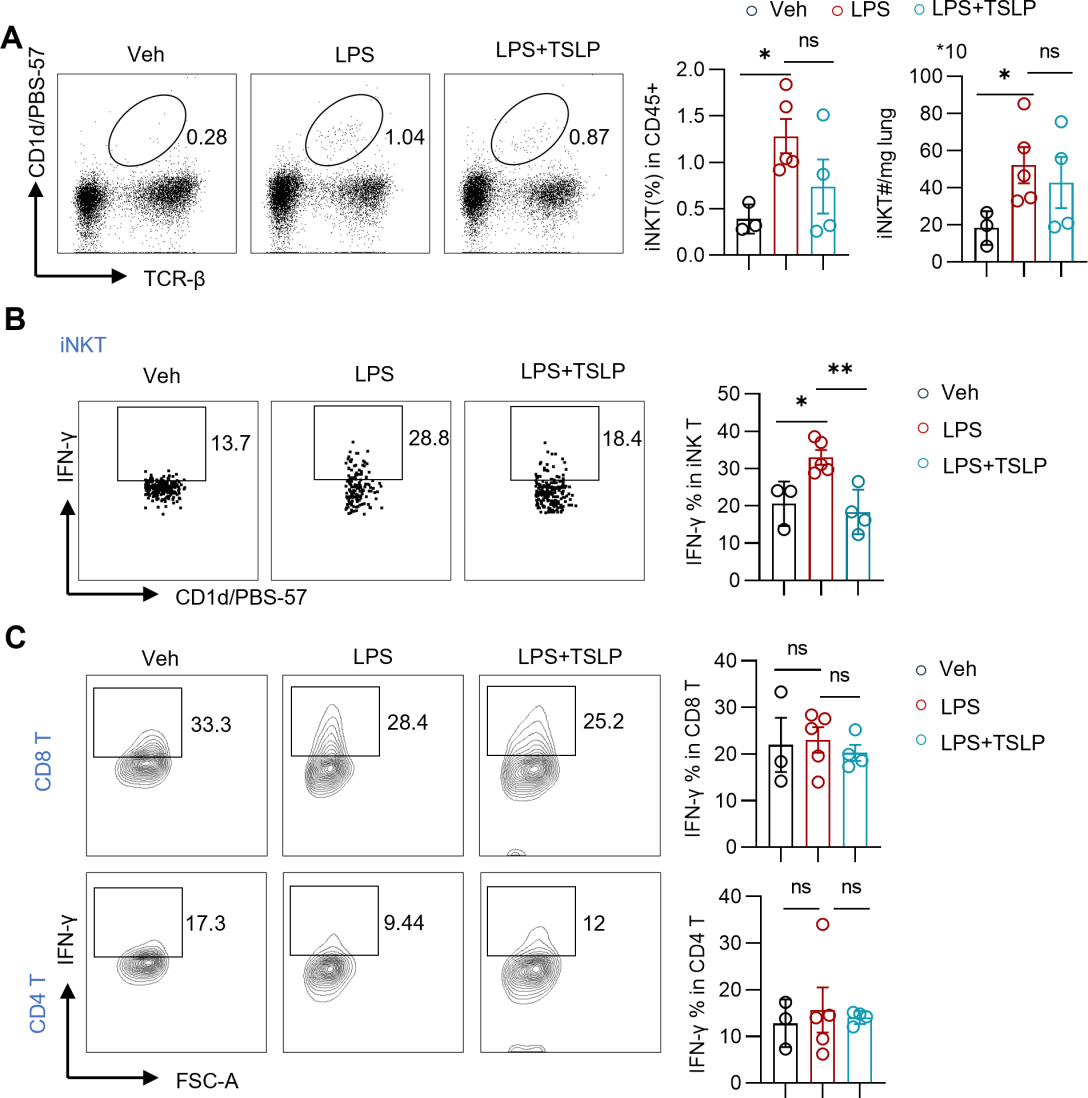
Exogenous TSLP suppresses IFN-γ-producing iNKT cells in LPS-induced acute lung injury. **(A)** Representative plots and summary bar graphs with scatter plots depicting ratio and counts of iNKT cells in lung tissues of indicated groups. n = 3 (Veh), n=5 (LPS), n=4 (LPS+TSLP), unpaired Student’s t test for statistics analysis between Veh group and LPS group or LPS group and LPS+TSLP group. **(B, C)** Representative plots and summary bar graphs with scatter plots depicting ratios of IFN-γ-producing iNKT cells, CD8 T cells, and CD4 T cells in indicated groups. n = 3 (Veh), n=5 (LPS), n=4 (LPS+TSLP), for ratios of IFN-γ-producing iNKT cells, Mann Whitney test for statistics analysis between Veh group and LPS group, unpaired Student’s t test for statistics analysis between LPS group and LPS+TSLP group. For ratios of IFN-γ-producing CD8 T cells and CD4 T cells, unpaired Student’s t test for statistics analysis between Veh group and LPS group or LPS group and LPS+TSLP group. *P < 0.05, **P < 0.01, ns, no significance. TSLP, Thymic Stromal Lymphopoietin, LPS, Lipopolysaccharide, Veh, Vehicle. Data are represented as mean ± SEM.

To further clarify which population of IFN-γ-producing cells was affected by TSLP, we detected the cell populations as shown in **Figure 4E**. Given that iNKT cells represent the major subset of innate-like T cells in mice, we assessed changes in pulmonary iNKT cells in an LPS-induced model with or without TSLP pretreatment. In contrast to the minimal changes observed in the ratio and number of conventional T cells (**Figure 2C**), the LPS group exhibited a significant increase in the proportion and number of iNKT cells in lung tissues compared to the Veh group (**Figure 5A**). Upon stimulation with PMA/Ion, pulmonary iNKT cells from the LPS group produced higher levels of IFN-γ than those from the Veh group (**Figure 5B**). Notably, while TSLP pretreatment did not affect the proportion or quantity of iNKT cells, it significantly attenuated the LPS-induced IFN-γ production capacity of these cells (**Figures 5A, B**). In contrast, no similar trend was observed for pulmonary CD4 T cells or CD8 T cells (**Figure 5C**). Meanwhile, there was a tendency to promote the secretion of IL-4 upon TSLP pretreatment (**Supplementary Figure 4A**). Taken together, these results indicate that TSLP suppresses IFN-γ production by iNKT cells, thereby identifying iNKT cells as a key target cell of TSLP.

### The role of iNKT cells in acute lung injury and their relationship with macrophage polarization

**Figure 6:**
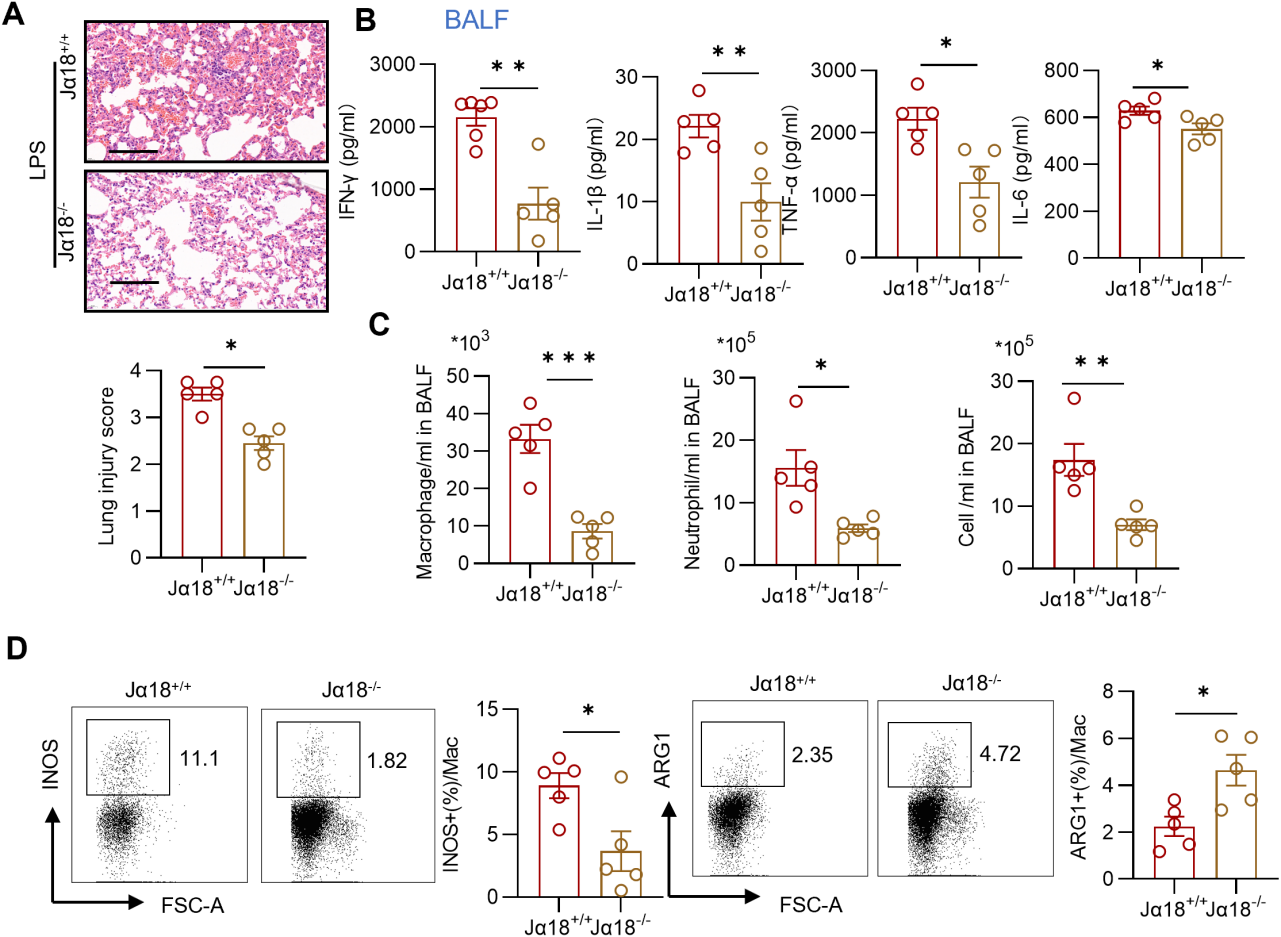
Depletion of iNKT cells diminishes lung inflammation and relates to reduced M1 macrophages. **(A)** Representative sections and lung injury score evaluation of H&E staining. Scale bar = 100μm. n = 5 per group, unpaired Student’s t test for statistics analysis between Jα18^+/+^ group and Jα18^-/-^ group. **(B)** Summary bar graphs with scatter plots showing the concentration of IFN-γ, TNF-α, IL-6, and IL-1β from BALF of indicated groups. n = 5 per group, unpaired Student’s t test for statistics analysis between Jα18^+/+^ group and Jα18^-/-^ group. **(C)** Summary bar graphs with scatter plots showing neutrophil counts, macrophage counts, and the total cell counts in BALF of indicated groups. n = 5 per group, unpaired Student’s t test for statistics analysis between Jα18^+/+^ group and Jα18^-/-^ group. **(D)** Representative plots and summary bar graphs with scatter plots depicting the ratio of INOS^+^ or ARG1^+^ pulmonary macrophages from indicated groups. n = 5 per group, unpaired Student’s t test for statistics analysis between Jα18^+/+^ group and Jα18^-/-^ group. *P < 0.05, **P < 0.01, ***P < 0.001. LPS, Lipopolysaccharide, BALF, Bronchoalveolar lavage fluid, Mac, macrophages. Data are represented as mean ± SEM.

To further elucidate the role of iNKT cells and their potential relationship with macrophages in acute lung injury, we analyzed inflammatory injury and infiltrating cells in LPS-treated Jα18^−/−^ mice, which are deficient in iNKT cells (19). Compared with wild-type mice, Jα18^−/−^ mice exhibited significantly attenuated lung injury and reduced production of IFN-γ, IL-6, TNF-α, and IL-1β production in BALF following LPS administration (**Figures 6A, B**). Moreover, the counts of macrophages, neutrophils, and total cells in the BALF of LPS-treated Jα18^−/−^ mice were greatly decreased compared to those in LPS-exposed wild-type mice (**Figure 6C**). This finding suggests that the absence of iNKT cells reduced infiltration of inflammatory cells into the lungs. Additionally, in iNKT-deficient mice, pulmonary macrophages exhibited a decreased proportion of the M1 phenotype, characterized by INOS expression, and an increased frequency of the M2 phenotype, marked by ARG1 expression (**Figure 6D**). Collectively, these results indicate that the absence of iNKT cells is associated with reduced lung inflammation and a skewing toward M2 macrophage differentiation in LPS-induced acute lung injury and suggests a potential link between iNKT cells and M1 macrophage polarization in acute lung injury.

## Discussion

In recent years, TSLP has garnered extensive attention for its involvement in various disorders, extending beyond its well-known role in allergies (20–23). Emerging studies have unveiled its protective role in nonalcoholic steatohepatitis (24), and acute murine graft-versus-host disease (25). Notably, Choa et al. (24) demonstrated that TSLP directly activates T cells in an antigen-independent manner, thereby offering protection against obesity and NASH. In the context of sepsis-induced liver injury and liver I/R injury, the hepatoprotective effects of TSLP have been confirmed through the activation of the PI3K/Akt pathway (26, 27). Additionally, TSLP has been shown to mitigate bleomycin-induced lung inflammation by reducing caspase-1 and caspase-3 activity in bronchial epithelial cells (28). However, despite these beneficial effects, TSLP’s role in inflammation remains paradoxical. Some studies suggest that TSLP may contribute to lung inflammation (29), highlighting the complexity of its function. This controversy surrounding endogenous TSLP’s role in lung inflammation has thus far limited our understanding of its impact on sepsis associated ARDS.

In this study, considering the dose (2μg/mouse, i.p.) as previously described (26), we pretreated mouse with TSLP at a concentration of 10 μg/kg intranasally and achieved the effect of reducing lung inflammation while the concentration of 2.5 μg/kg had no significant effect, indicating it might approach the minimum effective concentration for this model. While the intranasal dose may exceed physiological BALF concentrations (typically <20 pg/mL (30)), this pharmacological strategy ensures adequate target engagement in acute lung inflammation. This finding aligns with previous study that allergic immune diseases are associated with improved outcome in sepsis and pneumonia. While our study focuses on TSLP pretreatment, it is unclear that whether post-LPS or post-inflammatory-phase TSLP administration yield the same or divergent outcomes, which may bring different clinical significance. Our results provide evidence that TSLP may serve as a protective factor in acute inflammatory conditions, warranting further investigation into its therapeutic potential.

Innate immunity plays a crucial role in the early stages of sepsis associated ARDS, serving as the body’s first line of defense against pathogens. Macrophages, a vital component of innate immunity, exhibit remarkable diversity and plasticity during inflammatory response. Upon exposed to toll-like receptor (TLR) ligands and IFN-γ, resident macrophages become classically activated into the M1 phenotype, whereas stimulation by IL-4/IL-13 induces an alternative activation pattern (M2), mirroring the Th1/Th2 polarization of T cells (31). Pro-inflammatory M1 macrophages play a pivotal role in the exudative phase of ARDS by secreting a variety of Th1-cytokines such as TNF-α. Therefore, understanding factors that influence macrophage polarization and developing strategies to modulate M1 macrophage activation may be helpful to ARDS. In this study, we investigated that TSLP pretreatment on macrophage infiltration and polarization of M1 macrophages in LPS- induced acute lung injury. Notably, this effect was specific to macrophages, as TSLP pretreatment did not significantly alter the number or proportion of conventional CD8 T cells or CD4 T cells. These findings highlight the potential of TSLP as a targeted intervention to modulate macrophage polarization, thereby mitigating the inflammatory cascade in ARDS.

ARDS is characterized by a highly lethal inflammatory cascade, and the critical importance of early interventions cannot be overstated. However, the precise triggers of the inflammatory cytokine storm remain poorly understood. Studies using pneumonic ARDS models have demonstrated that IFN-γ signaling can elicit an exaggerated response from myeloid cells, leading to an overwhelming inflammatory cytokine storm and subsequent lung tissue damage (32–34). For instance, in mouse models of methicillin-resistant *Staphylococcus aureus* (MRSA) pneumonia, the combination of IFN-γ deficiency and antibiotic therapy significantly improves survival rates compared to antibiotic therapy alone (34). Furthermore, *Ifng* knockout has been shown to reduce neutrophil chemotaxis, thereby protecting against lung injury induced by excessive oxygen exposure (35). In this study, we identified IFN-γ as a potential target of TSLP. Our findings suggest that the protective effect of TSLP in inhibiting neutrophil chemotaxis and macrophage cytokine production are likely mediated through modulation of IFN-γ signaling. This highlights the potential role of TSLP in mitigating the inflammatory cascade associated with acute lung injury.

iNKT cells, a subset of IFN-γ-producing cells, are known for their rapid response to external stimuli (36) and their potent immunological modulatory functions (37). As a key component of unconventional T cells, iNKT cells exhibit both innate and adaptive properties. They express relatively restricted T cell receptors and recognize glycolipid antigens presented by CD1d molecule, which is expressed on macrophages and other antigen-presenting cells (38). In co-culture systems involving iNKT cells and macrophages, neutralizing of IFN-γ has been shown to reduce the expression of pro-inflammatory macrophage-related genes while increasing the expression of genes associated with reparative macrophages (39). Consistent with these findings, our study indicates that IFN-γ-producing iNKT cells contribute to LPS-induced acute lung injury, likely through their association with the polarization of proinflammatory M1 macrophages.

Here, our study elucidated the protective effect of TSLP in LPS-induced acute lung injury. This effect involves the regulation of the reprogramming of IFN-γ-producing iNKT cells. Acting as early responders in the immune environment, iNKT cells can regulate the downstream pro-inflammatory phenotype of macrophages and the recruitment of neutrophils via IFN-γ, ultimately mediating the regulation of inflammation. Nevertheless, the specific connections require further clarification.

## Conclusions

Overall, our study underscores the crucial role of TSLP in sepsis-associated ARDS and provides valuable insights supporting TSLP as a potential beneficial predictor. Moreover, it provides an explanation for the phenomenon that allergic immune diseases are associated with an improved prognosis in sepsis and pneumonia.

## Data Availability

The Bulk RNA-seq data GSE262107 can be accessed from the NCBI Gene Expression Omnibus (GEO).
