# Supplementary figures and images for: TSLP pretreatment inhibits M1 macrophage polarization and attenuates LPS-induced iNKT cell-dependent acute lung injury
Source: Front Immunol. 2025 May 23;16:1583235. doi: 10.3389/fimmu.2025.1583235 (PMC12141294; doi:10.3389/fimmu.2025.1583235)

SUP Fig 1 :

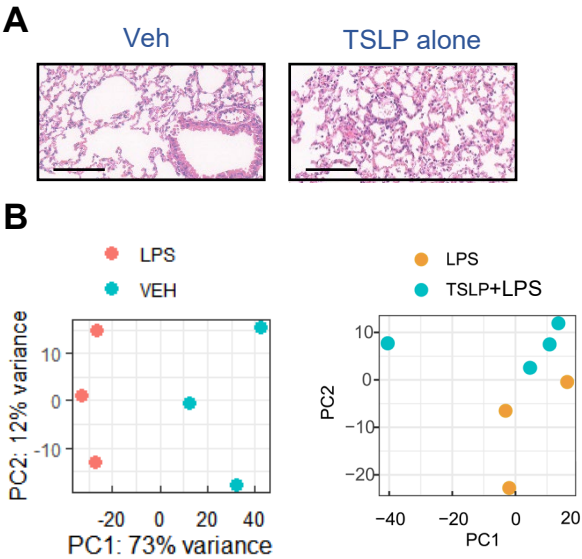

SUP Fig 2 :

**A**

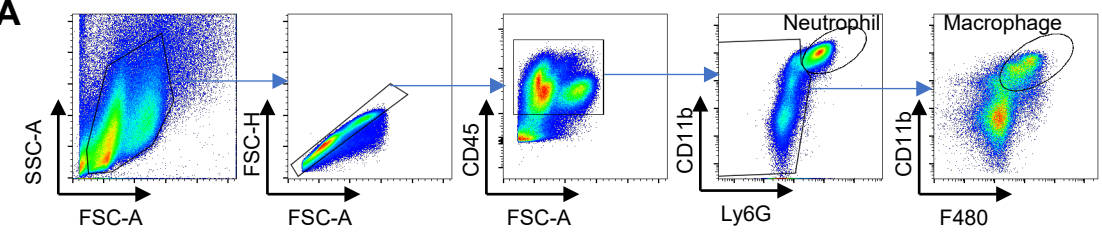

**B**

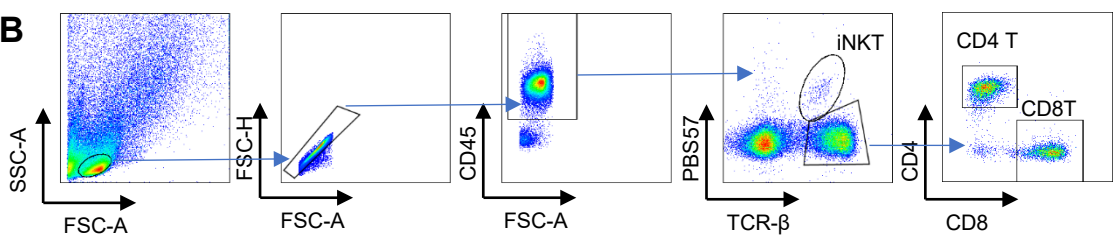

SUP Fig 3 :

**A**

GSE167118

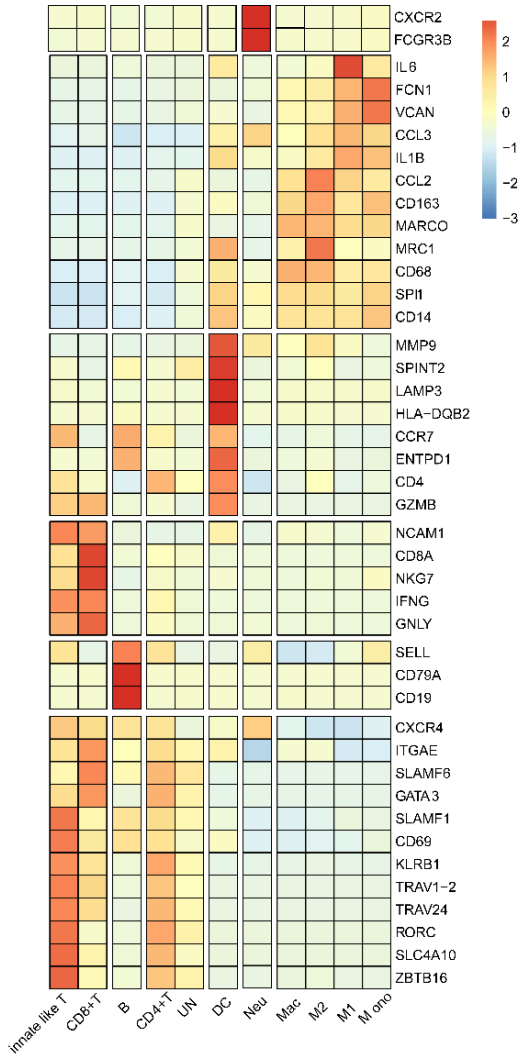

SUP Fig 4 :

**A**

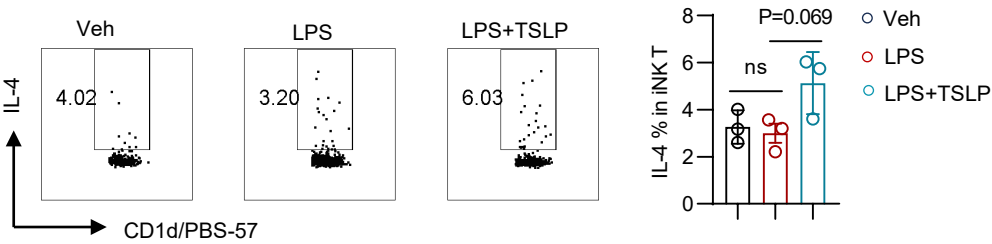

Supplement: Supplementary file 1 [file Image1.pdf]
